# Thysanoptera of Bulgaria

**DOI:** 10.3897/zookeys.504.9576

**Published:** 2015-05-19

**Authors:** Olia Karadjova, Vladimir Krumov

**Affiliations:** 1 Institute of Soil Science, Agrotechnology and Plant Protection, 7 Shosse Bankya str., Sofia 1080, Bulgaria

**Keywords:** Thysanoptera, Bulgaria, checklist, geographic distribution, feeding preference

## Abstract

The present checklist includes data on the species composition, geographic distribution and feeding preferences of thrips species in Bulgaria. In total, 155 species in 48 genera are listed. Of these, 125 species belong to suborder Terebrantia and include 103 species of 33 genera in family Thripidae, 14 species of two genera in Aeolothripidae, seven species of two genera in Melanthripidae and one species in Fauriellidae. In suborder Tubulifera, 30 species of 10 genera in the single family Phlaeothripidae are listed. Of the 155 Bulgarian thrips species, 87.7% are phytophagous, 4.5% are obligate predators, 5.8% are mycophagous and 1.9% are with unknown feeding preferences. Fourteen pest species are listed for Bulgaria, of which *Frankliniella occidentalis*, *Thrips tabaci* and *Haplothrips tritici* are of economic importance. The list provides detailed information on the horizontal and vertical distribution of Thysanoptera in 5 regions and 45 subregions of Bulgaria. The present paper also includes an evaluation of the biodiversity of Thysanoptera and the extent to which each region of the country has been studied.

## Introduction

Bulgaria is located on the Balkan Peninsula and extends from the western shore of the Black Sea to Serbia and Macedonia to the west. It lies in the transitional area between the contrasting continental and Mediterranean climatic zones. Its varied relief and the peculiar characteristics of its weather contribute to its biotope diversity. According to the Palaearctic classification (Devillers et al. 2001), 977 distinct habitats from all hierarchical types occur in Bulgaria, 96 of which are unique to the country. This richness of habitats on a relatively small area is a prerequisite for a diverse thysanopteran fauna.

At present, the Bulgarian entomofauna is insufficiently studied and it has been estimated that about 51% of the insect species are known. More than 29 000 species of superclass Hexapoda have been established and it is expected that if rigorous research is performed, their number would increase to 56 000 (Hubenov 2005).

Thrips are small and slender insects that generally feed on plant sap, fungal spores and some on them are predators of small arthropods. Until now about 6000 species have been described worldwide (ThripsWiki 2015). Some are pests of agricultural crops and ornamentals, causing damage to plants either by feeding or via transmission of plant viruses, pathogenic bacteria and fungi.

The biodiversity of thrips on the Balkans has been studied more extensively in Romania (Vasiliu-Oromulu 1998) and Serbia (Andjus et al. 2008) with 215 and 155 reported species respectively. Information is scarce on the thysanopteran fauna in the other neighbours of Bulgaria: Greece, Macedonia and the European part of Turkey. After considering the climate and the number of described species of superclass Hexapoda in Bulgaria’s neighbours and on the European continent as a whole, Hubenov (1996) claimed that there should be about 250 species of thrips in the country.

The thysanopterological activities in Bulgaria began at the end of the 19^th^ century. The first thrips species recognised in the country, *Thrips urticae*, was recorded on tobacco (Manushev 1897). Malkov recorded *Limothrips cerealium* and a year later *Thrips tabaci* (Malkov 1902, 1903), and *Limothrips denticornis* was recorded on rye and barley (Dospevski 1910). Following these, *Haplothrips reuteri*, *Aeolothrips fasciatus*, *Haplothrips tritici*, *Heliothrips haemorrhoidalis* and *Thrips atratus* were recorded (Chorbadjiev 1929). In 1958, the Czech entomologist Pelikán conducted a study of the Bulgarian thrips fauna. He was the first to report 13 species of Aeolothripidae, described a new genus and species of Fauriellidae, *Ropotamothrips buresi*, and two years later recorded *Melanthrips paspalevi* and *Melanthrips titschacki* from Bulgaria (Pelikán 1960a, 1960b).

In the late 60’s, thrips research became more active. Janev (1968 and 1973) reported 22 species. In 1967, Genov reported two *Haplothrips* species on alfalfa. Donchev (1968, 1972, 1976, 1984, 1993 and 1996) contributed to the Bulgarian thrips fauna with a series of publications, recording 33 species. Vesselinov (1968, 1976) recorded eight species; and. Popov (1973) recorded four thrips species found on medicinal plants which were new to the fauna of Bulgaria. Moreover, Popov (1976; 1982a, 1985; 1988) carried out extensive research on the diversity of thrips in Ograzhden mountain and reported 22 further species. He was also the first to document the Bulgarian tree-living thysanopteran fauna, reporting another 13 species from the country (Popov 1982b). Schliephake (1982) reported *Thrips fedorovi* from this country.

Trenchev (1991) reported *Frankliniella occidentalis* in Bulgaria, and Trenchev and Karadjova (1992) reported its distribution and host plants in Bulgarian greenhouses. In 1996, after revision of microscope slides, the record of *Anaphothrips armatus* was cancelled because the reference specimens turned out to represent *Rubiothrips ferrugineus* (Zur Strassen 1996). *Echinothrips americanus* was first reported in 2003 by Karadjova and Krumov. A recent contribution to the arboreal thrips fauna of Bulgaria is the report of the mulberry thrips, *Pseudodendrothrips mori*, on the leaves of *Morus alba* (Trenchev and Trencheva 2007). Jenser and Krumov (2009) newly reported nine species. Krumov (2013) reported *Idolimothrips paradoxus* and *Iridothrips iridis* for the first time for the fauna of Bulgaria.

The main aim of this paper is to summarize all published data on thrips from Bulgaria in order to present a full list of known thrips taxa from the entire area of the country. Until now, no comprehensive review of the Bulgarian thrips fauna has been published. The present list includes 125 species of suborder Terebrantia and 30 species of suborder Tubulifera, and provides detailed information on the horizontal and vertical distribution of Thysanoptera in Bulgaria. It is complemented by an evaluation of the biodiversity and the extent to which each region of the country has been studied. The territorial distribution of thysanopteran species is crucial for the understanding of their biology and adaptations to different habitats. Such knowledge is of basic importance to explain the introduction and spread of exotic species, particularly pest species (Marullo and Grazia 2013). Another aim of the paper is to present information on the feeding preferences of thrips in Bulgaria: whether they are predatory, mycophagous or phytophagous and what plant species they have been collected from. This is important in order to understand the role of thrips in ecosystems, to ascertain which plants support phytophagous thrips (Mound and Marullo 1996), to evaluate their pest potential and to assess the impact of different thrips species on populations of other organisms within crops and natural non-cultivated areas

## Material and methods

The list was prepared after a thorough review of all available publications and individual samples collected by the authors. The review includes all 37 scientific papers on the thysanopteran fauna in Bulgaria, published from 1897 to 2013. The list is arranged systematically and the nomenclature follows ThripsWiki (2015). Genera are listed alphabetically within each family or subfamily, and species are similarly listed within each genus. Each species account includes its taxonomic name, references, locality records within Bulgaria, altitudinal range (in m.a.s.l), plants on which adults have been found, and whether predatory, mycophagous or phytophagous. The geographic regions of Bulgaria and their abbreviations used in the text follow the division of Hubenov (1997), developed for the purposes of faunistic research. It does not consider the administrative territories but rather uses characteristics such as relief and local climatic conditions. The division includes five major territories, further split into subregions. The subheading “Distribution” for each species refers only to localities within Bulgaria.

B Black Sea Coast:

BN Northern Black Sea Coast,

BS Southern Black Sea Coast.

D Danubian Plain:

DE Eastern Danubian Plain:

DEL Ludogorie–Dobrudja District,

DEP Popovo–Provadiya District,

DM Middle Danubian Plain,

DW Western Danubian Plain,

P Transitional Region:

PB Tundja–Strandja Subregion:

PBB Bakadjik–Burgas District,

PBC Sakar Mts.,

PBD Strandja–Dervent District,

PBS Strandja Mts,

PBT Sakar–Tundja District

PK Kraishte–Konyavo District:

PKG Golo Bardo Mts.,

PKK Kraishte,

PKQ Konyavska Planina Mts.,

PKR Rui Mts.,

PKV Verila Mts.,

PKZ Zemenska Planina Mts.

PS Srednogorie–Podbalkan Subregion:

PSA Sredna Gora,

PSC Sashtinska Sredna Gora Mts.,

PSI Ihtimanska Sredna Gora Mts.,

PSL Lozenska Planina Mts.,

PSP Podbalkan Basins,

PSS Sredna Gora Mts.

PT Thracian Lowland

PV Vitosha District:

PVL Lyulin Mts.,

PVP Plana Mts.,

PVS Sofia Basin,

PVV Vitosha Mts.,

PVW Viskyar Mts.

R Rila–Rhodope Massif:

RO Osogovo–Belasitsa Group:

ROB Belasitsa Mts.,

ROG Ograzhden Mts.,

ROM Maleshevska Planina Mts,

ROO Osogovska Planina Mts.,

ROP Krupnik–Sandanski–Petrich Valley,

ROS Srednostrumska Valley,

ROT Boboshevo–Simitli Valley,

ROV Vlahina Planina Mts.

RP Rila–Pirin Group:

RPM Mesta Valley,

RPP Pirin Mts.,

RPR –Rila Mts.,

RPS Slavyanka Mts.,

RPT Stargach Mts.

RR Rhodope Mts.:

RRE Eastern Rhodope Mts.,

RRW Western Rhodope Mts.

S Stara Planina Range:

SP Predbalkan (Pre-Balkan or foothills north of Stara Planina Mts.):

SPW Western Predbalkan,

SPM Middle Predbalkan,

SPE Eastern Predbalkan

SB Stara Planina (Balkan) Mts:

SBW Western Stara Planina Mts.,

SBM Middle Stara Planina Mts.,

SBE Eastern Stara Planina Mts.

## Suborder Terebrantia Haliday

Four families of this suborder are recorded from Bulgaria: Aeolothripidae, Melanthripidae, Fauriellidae and Thripidae. Thripidae is the largest family and includes the most economically important pest species.

### Family Aeolothripidae Uzel

The family includes 190 extant species in 23 genera worldwide (ThripsWiki 2015). The adults and larvae of many representatives of this family appear to be facultative predators of small arthropods, although some species are almost certainly solely phytophagous (Tyagi et al. 2008). In the warmer parts of the world, a considerable number of species in family Aeolothripidae are obligate predators (Hoddle 2003). In Bulgaria, 14 species belonging to two genera have been recorded.

***Aeolothrips albicinctus* Haliday, 1836**

**Distribution.** **DM** – Krushovitsa (160 m). Obligate predator residing at the collar of grasses, collected from *Festuca aerudinacea* (Donchev and Tomov 1996).

***Aeolothrips astutus* Priesner, 1926**

**Distribution.** **DM** – Krushovitsa; **ROP** – Kresna, Parvomai, Petrich, Samuilova krepost; **RPR** – Rila Monastery (150–300 m). Phytophagous and facultative predator, collected from *Anchusa* sp., *Echium vulgare*, different grasses (Pelikán 1958, Donchev 1968, Popov 1982a).

***Aeolothrips balati* Pelikán, 1958**

**Distribution.** **RPP** – Pirin (below Banderitsa) (1600 m). Predator, found in alpine meadows (Pelikán 1958).

***Aeolothrips collaris* Priesner, 1919**

**Distribution.** **BN** – Obzor; **BS** – Primorsko, Rosen, Ropotamo; **DM** – Krushovitsa; **PVP** – Pancharevo; **ROB** – Belasitsa; **ROP** – Samuilova krepost; **ROT** – Simitli; **RPP** – Banderitsa; **RPR** – Rila Monastery; **SBE** – Sinite Kamani (0–1810 m). Phytophagous and facultative predator, collected from *Achillea compacta*, *Alyssum montanum*, *Brassicaceae* species, *Campanula* sp., *Castanea sativa*, *Clematis vitalba*, *Colutea arborescens*, *Coronilla varia*, *Euphorbia* sp., *Medicago sativa*, *Onobrychis sativa*, *Paliurus aculeatus*, *Senecio* sp., *Symphytum* sp., *Trifolium pratense* (Pelikán 1958, Donchev 1976, Popov 1976, 1982a).

***Aeolothrips ericae* Bagnall, 1920**

**Distribution.** **BS** – Ropotamo; **PBC** – Topolovgrad; **PSP** – Sliven; **PVP** – Pancharevo; **ROG** – Karnalovo; **ROP** – Parvomai, Petrich; **ROT** – Blagoevgrad; **RPP** – Banderitsa; **RPR** – Partizanska poliana, Rila Monastery; **RRW** – Chaira, Smolyan lakes; **SBW** – Lakatnik (0–1810 m). Phytophagous and facultative predator, collected from *Astragalus* sp., *Coronilla emerus*, *Coronilla varia*, *Daphne cneorum*, *Daphne oleoides*, *Fabaceae* species (flowers), *Lotus corniculatus*, *Syringa vulgaris*, *Tropaeolum majus*, grassy vegetation in forests (Pelikán 1958, Yanev 1973, Donchev 1976, Popov 1976, 1982a).

***Aeolothrips fasciatus* (Linnaeus, 1758)**

**Distribution.** **DEL** – Ruse, Obrazov chiflic; **PVL** – Lyulin Monastery; **PVS** – Sofia basin; **ROG** – Karnalovo, Nikudin; **ROP** – Petrich, Samuilova krepost; **ROT** – Blagoevgrad; Rila; **RP** – Predela – Gradevo; **RPP** – Dolnoto breznichko ezero; **RPR** – Ribni ezera; **RRW** – Golyam Beglik dam (100–2230 m). Phytophagous and facultative predator, collected from *Brassica napus*, *Cannabis sativa*, *Cytisus* sp., *Helianthus annuus*, *Nicotiana tabacum*, *Rosa* sp., *Trifolium pratense*, *Zea mays*, grasses and shrubs in forests (Chorbadzhiev 1929, Yanev 1973, Popov 1976, 1982a, 1982b).

***Aeolothrips gloriosus* Bagnall, 1919**

**Distribution.** **ROP** – Kresna (170 m). Phytophagous and facultative predator, collected from *Clematis vitalba* (Jenser and Krumov 2009).

***Aeolothrips intermedius* Bagnall, 1934**

**Distribution.** **BS** – Mandra lake, Primorsko; **DM** – Pleven – Chaira, Krushovitsa; **PBB** – Karnobat; **PT** – Stara Zagora; **PVP** – Pancharevo; **PSL** – Gorni Lozen; **PVS** – Kostinbrod, Opitsvet, Svetovrachane; **ROB** – Belasitsa Mts.; **ROP** – Petrich; **ROT** – Simitli; **RPP** – Banderits); **RPR** – Rila Monastery; **SBE** – Sinite kamani; **SBM** – Beklemeto, Troyan (0–1810 m). Phytophagous and facultative predator, collected from *Beta vulgaris*, *Campanula* sp., *Echium vulgare*, *Galium* sp., *cereals*, *Hordeum vulgare*, *Lathyrus sativus*, *Lotus corniculatus*, *Medicago sativa*, *Melilotus officinalis*, *Onobrychis sativa*, *Sinapis arvensis*, *Solanum dulcamara*, *Soja hispida*, *Trifolium incarnatum*, *Trifolium repens*, vegetation of grasses and shrubs (Pelikán 1958, Genov 1967, Donchev 1968, 1972, Yanev 1973, Popov, 1982a).

***Aeolothrips melaleucus* Haliday, 1852**

**Distribution.** **DM** – Pleven-Chaira; **PVP** – Pancharevo; **PSP** – Sliven; **ROG** – Churicheni, Markovi Kladentsi (150–1530 m). Obligate predator, collected from leaves of *Castanea sativa*, *Crataegus* sp., *Fraxinus* sp, *Ligustrum* sp., *Quercuss* sp., *Sambucus* sp., *Solanum dulcamara*, *Sorbus* sp. (Pelikán 1958, Donchev 1968, Popov 1982a).

***Aeolothrips priesneri* Knechtel, 1923**

**Distribution.** **PVP** – Pancharevo (600–800 m). Unknown feeding preferences, collected from *Euphorbia* sp. (flowers) (Pelikán 1958).

***Aeolothrips propinquus* Bagnall, 1924**

**Distribution.** **BS** – Ropotamo; **ROP** – Petric); **ROT** – Simitli; **RPR** – Rila Monastery (0–1150 m). Phytophagous and facultative predator, collected from *Anchusa officinalis* (Pelikán 1958).

***Aeolothrips versicolor* Uzel, 1895**

**Distribution.** **BS** – Ropotamo; **ROB** – Belasitsa Mts.; **RPR** – Rila Monastery (0–1150 m). Obligate predator, collected from leaves of *Castanea sativa* (Pelikán 1958).

***Aeolothrips vittatus* Haliday, 1836**

**Distribution.** **ROG** – Ograzhden Mts. (100–1530 m). Predator of arthropods, collected from *Pinus* sp. (Popov 1985).

***Rhipidothrips gratiosus* Uzel, 1895**

**Distribution.** **DM** – Krushovitsa; **PBB** – Karnobat; **PK** – Breznik valley; **PVS** – Prolesha, Svetovrachane (170–760 m). Phytophagous (Marullo and Grazia 2013) and facultative predator (Bailey 1954), collected from *Avena sativa*, *Hordeum vulgare*, *Onobrychis sativa*, *Triticum aestivum* (Donchev 1968, 1972, 1976, Yanev 1973).

### Family Melanthripidae Bagnall

This family includes 65 species of four genera. All representatives feed on flowers, but the distribution of the genera is remarkably fragmented (ThripsWiki 2015). In Bulgaria, 7 species of two genera have been recorded.

***Ankothrips niezabitowskii* (Schille, 1910)**

**Distribution.** **PVV** – Cherni vrah; **SBW** – Belidie han (735–2290 m). Phytophagous, collected from *Juniperus communis*, *Juniperus procumbens* (Popov 1982b).

***Melanthrips acetosellae* John, 1927**

**Distribution.** **BS** – Ropotamo; **RPR** – Rila Monastery; **PSP** – Sliven (0–1150 m). Unknown feeding preferences (Pelikán 1958).

***Melanthrips fuscus* (Sulzer, 1776)**

**Distribution.** **DEP** – Makariopolsko; **DM** – Krushovitsa; **PVS** – Suhodol; **PVP** – Pancharevo; **ROG** – Churicheni; **ROP** – Petrich; **RPM** – Bany); **SBE** – Sinite kamani (155–1000 m). Phytophagous, collected from *Onobrychis sativa*, *Rosa* sp., *Sinapis arvensis*, *Syringa vulgaris*, grassy vegetation (Pelikán 1958, Yanev 1973, Donchev 1976, Popov 1982a, 1982b).

***Melanthrips knechteli* Priesner, 1936**

**Distribution.** **BS** – Ropotamo (0 m). Phytophagous, collected from forest and steppe vegetation (Pelikán 1958).

***Melanthrips pallidior* Priesner, 1919**

**Distribution.** **BS** – Mandra lake, Primorsko, Rosen, Ropotamo; **DEP** – Makariopolsko; **DM** – Krushovitsa, Obnova, Pordim, Vulchi trun; **DW** – Vidin; **PVP** – Pancharevo; **PVS** – Opitsvet, Trebich; **ROG** – Churicheni; **ROP** – Petrich; **RPP** – Banderitsa; **RPR** – Borovec, Rila Monastery; **SBE** – Sinite kamani; **SBW** – Lakatnik; **SPW** – Botevgrad (0–1810 m). Phytophagous, collected from *Agrostemma githago*, *Campanula* sp., *Colutea arborescens*, *Coronilla varia*, *Cruciferous* species, *Echium vulgare*, *Latirus tuberosus*, *Lotus corniculatus*, *Medicago sativa*, *Onobrychis sativa*, *Symphytum* sp., *Trifolium incarnatum*, *Trifolium repens*, *Veronica spicata*, blooming grasses (Pelikán 1958, Donchev 1968, 1972, 1976, Yanev 1973, Popov 1982a).

***Melanthrips paspalevi* Pelikán, 1960**

**Distribution.** **SBE** – Sinite kamani; **SBW** – Lakatnik (550–950 m). Phytophagous, collected from steppe vegetation (Pelikán 1960b).

***Melanthrips titschacki* Pelikán, 1960**

**Distribution.** **BS** – Ropotamo; **PVP** – Pancharevo; **ROB** – Belasitsa Mts.; **RPP** – Banderitsa; **SBE** – Sinite kamani (0-1810 m). Phytophagous, collected from steppe and forest vegetation, mixed populations with *Melanthrips pallidior* (Pelikán 1960a).

### Family Fauriellidae Priesner

Five species belonging to four genera have been described worldwide but very little is known about them (ThripsWiki 2015). In Bulgaria, only one species has been reported.

***Ropotamothrips buresi* Pelikán, 1958**

**Distribution.** **BS** – Ropotamo (0 m). Unknown feeding preferences (Pelikán 1958) According to zur Strassen (2003) *Ropotamothrips buresi* is possibly associated with *Artemisia*.

### Family Thripidae Stephens

This family includes 1970 species in 287 genera worldwide, systematized in four subfamilies: Dendrothripinae, Panchaetothripinae, Sericothripinae and Thripinae (ThripsWiki 2015). Most of the species are phytophagous (Mound 2002), but a few are obligate predators (Mound 2011). *Frankliniella occidentalis* and *Thrips tabaci* are polyphagous pests but also behave as facultative predators in some regions (Wilson et al. 1996), and the genus *Aulacothrips* includes five species that are ectoparasitic on Hemiptera (Cavalleri et al. 2013, Cavalleri and Kaminski 2014). In Bulgaria, 103 species from 33 genera have been recorded.

#### Subfamily Dendrothripinae Priesner

***Dendrothrips degeeri* Uzel, 1895**

**Distribution.** **ROB** – Belasitsa; **ROG** – Churicheni; **ROP** – Kulata, Petrich; **RPP** – Pirin Mts.; **RPR** – Rila Mts. (85–1490 m). Phytophagous, collected from *Abies alba*, *Corylus avellana*, *Fagus sylvatica*, *Morus alba*, *Ostrya carpinifolia* (Popov 1985, 1988).

***Dendrothrips ornatus* (Jablonowski, 1894)**

**Distribution.** **PVS** – Sofia; **ROB** – Belasitsa Mts.; **ROP** – Petrich; **ROT** – Blagoevgrad; **RPP** – Pirin Mts.; **RPR** – Rila Mts.; **SPW** – Belidie han (360–1490 m). Phytophagous, plant pest, collected from *Alnus alba*, *Alnus incana*, *Syringa* sp., *Syringa vulgaris*, *Tilia* sp. (Veselinov 1976, Popov 1982b, 1988).

***Dendrothrips phillireae* (Bagnall, 1927)**

**Distribution.** **ROP** – Damyanitsa (120 m). Phytophagous, found on *Phillyrea media* (Popov 1982b).

***Dendrothrips saltator* Uzel, 1895**

**Distribution.** ROG – Divechova polyana; ROP – Sandanski (270-1150m). Phytophagous, found on *Alnus alba*, *Tamarix* sp. (Popov 1982a, 1982b).

***Pseudodendrothrips mori* (Niwa, 1908)**

**Distribution.** **DW** – Butan (60 m). Phytophagous, pest species on leaves of *Morus alba* (Trenchev and Trencheva 2007).

#### Subfamily Panchaetothripinae Bagnall

***Heliothrips haemorrhoidalis* (Bouche, 1833)**

**Distribution.** glasshouses: **PT** – Plovdiv, Pazardjik; **PVS** – Sofia basin; **ROP** – Petrich; **ROT** – Blagoevgrad. Phytophagous, pest of *Cucumis sativus*, leaves of ornamentals – *Citrus* sp., *Cyclamen* sp., *Fuchsia* sp., *Orchis* sp., *Rhododendron* sp. (Chorbadzhiev 1929, Elenkov and Hristova 1974, Atanasov et al. 2005).

#### Subfamily Sericothripinae Karny

***Neohydatothrips abnormis* (Karny, 1910)**

**Distribution.** **DM** – Komudara, Krushovitsa; **DEL** – Obrazov chiflic; **PBB** – Yambol; **ROG** – widespread in Ograzhden Mts.; **RPR** – Borovets, Musala peak; **SPM** – Gorsko Slivovo (160 – 2925 m). Phytophagous, collected from *Lotus corniculatus*, *Medicago sativa*, *Trifolium* sp., *Vicia* sp. (Donchev 1976, Popov 1982a).

***Neohydatothrips gracilicornis* (Williams, 1916)**

**Distribution.** **DM** – Krushovitsa; **PBB** – Karnobat; **PT** – Plovdiv; **ROG** – widespread in Ograzhden Mts.; **ROB** – Drangovo; **ROP** – Kresna, Samuilovo; **RPM** – Banichan; **RPP** – Predela – Gradevo, Dobrinishte (130 – 845 m). Phytophagous, collected from flowers of *Medicago sativa*, *Onobrychis sativa*, *Prunus* sp., *Prunus mahaleb*, *Prunus spinosa*, *Quercus petraea*, *Quercus* sp., *Soja hispida*, *Vicia* sp. (Donchev 1968, 1976; Popov 1976, 1982a, 1982b).

***Sericothrips bicornis* (Karny, 1910)**

**Distribution.** **ROG** – widespread Ograzhden Mts. (200–1000 m). Phytophagous, collected from *Lotus corniculatus*, *Trifolium* sp., *Vicia* sp. (Popov 1982a).

***Sericothrips staphylinus* Haliday, 1836**

**Distribution.** **PVV** – Bistritsa, Ostrica, Shevovitsa, Zheleznitsa; **ROG** – Karnalovo, Nikudin, Dolene (150–1640 m). Phytophagous, collected from *Bromus arvensis*, *Corylus avellana*, *Festuka elatior*, *Oxalis* sp., *Prunus cerasus*, *Prunus communis*, *Prunus domestica*, *Prunus persica*, *Prunus sativa*, *Prunus spinosa* (Yanev 1968, Popov 1982a, 1982b).

#### Subfamily Thripinae Stephens

***Anaphothrips euphorbiae* Uzel, 1895**

**Distribution.** **ROG** – Churicheni, Divechova polyana; **SBM** – Beklemeto, Troyan (300–1360 m). Phytophagous, collected from *Euphorbia rupestris*, *Euphorbia myrsinites*, *Galium* sp. (Donchev 1968, Popov 1982a).

***Anaphothrips obscurus* (Muller, 1776)**

**Distribution.** **DM** – (Krushovitsa); **PVP** – Pancharevo; **PT** – Pazardzhik; **PVV** – Simeonovo; **ROG** – widespread in Ograzhden Mts.; **ROT** – Blagoevgrad; RPP – Predela) (160–1000 m). Phytophagous, collected from *Avena sativa*, *Holcus lanatus*, *Hordeum* sp., *Medicago* sp., *Onobrychis sativa*, *Trifolium pratense*, *Triticum aestivum*, mixed Poaceae (Veselinov 1968, Donchev 1976, Popov 1976, 1982a).

***Aptinothrips elegans* Priesner, 1924**

**Distribution.** **PVS** – Obelya (500 m). Phytophagous, collected from *Triticum aestivum* (Veselinov 1968).

***Aptinothrips rufus* Haliday, 1836**

**Distribution.** **PVV** – Dragalevci; **PVP** – Pancharevo; **ROG** – Churicheni, Divechova polyana; **ROP** – Samuilova krepost (150–1150 m). Phytophagous, collected from *Avena sativa*, *Bromus* sp., *Hordeum* sp. (Veselinov 1968, Popov 1982a).

***Aptinothrips stylifer* Trybom, 1894**

**Distribution.** **RRW** – Studenets, Rock bridges (1450–1735 m). Phytophagous, collected from *Agrostis* sp., *Alopecurus* sp., *Dactylis glomerata* (Donchev 1993).

***Asphodelothrips croceicollis* (Karny, 1914)**

**Distribution.** **ROG** – Divechova polyana, Dolene, Markovi kladentsi (400–1535 m). Phytophagous, collected from mixed grass vegetation (Popov 1982a).

***Belothrips morio* O. M. Reuter, 1899**

**Distribution.** **PVV** – Kumata, Sredec, Selimitsa; **SBW** – Kom, Vezhen (100–1650 m). Phytophagous, collected from *Gnaphalium* sp., *Pinus montana*, *Rubus idaeus*, *Thymus* sp. (Yanev 1968, Popov 1982b).

***Bregmatothrips dimorphus* (Priesner, 1919)**

**Distribution.** **ROG** – Ograzhden Mts. (400–1000 m). Phytophagous, collected from mixed herbaceous vegetation (Jenser and Krumov 2009).

***Chirothrips aculeatus* Bagnall, 1927**

**Distribution.** **PVS** – Gorublyane (550 m). Phytophagous, collected from *Avena sativa* (Veselinov 1968).

***Chirothrips manicatus* Haliday, 1836**

**Distribution.** DM – Krushovitsa; **DEL** – Obraztsov chiflik; **PT** – Plovdiv; **PVP** – Pancharevo; **PVS** – Sofia; **PVV** – Dragalevci, Selimitsa; **ROG** – Nikudin; **SBM** – Beklemeto (130–1360 m). Phytophagous, collected from *Dactylis glomerata*, *Galium* sp., *Lotus corniculatus*, *Medicago sativa*, *Onobrychis sativa*, *Secale cereale*, *Solanum tuberosum*, mixed herbaceous vegetation (Veselinov 1968, Donchev 1968, 1976, Yanev 1973, Popov 1982a, 1982b).

***Chirothrips pallidicornis* Priesner, 1925**

**Distribution.** **RRW** – Rock bridges (1450 m). Phytophagous, collected from *Dactylis glomerata*, *Silene* sp. (Donchev 1993).

***Dictyothrips betae* Uzel, 1895**

**Distribution.** **PVV** – Aleko, Bistritsa, Dragalevci, Kupena, Rodina, Zheleznitsa; **ROG** – Nikudin; **ROP** – Purvomai, Samuilova krepost (150–1840 m). Phytophagous, collected from *Gnaphalium supinum*, *Juniperus* sp., *Melissa officinalis*, *Rosa* sp., *Salvia glutinosa*, *Silene juvenalis*, *Verbascum blattaria*, mixed herbaceous vegetation (Yanev 1968, Popov 1982a).

***Drepanothrips reuteri* Uzel, 1895**

**Distribution.** **SBW** – Berkovitsa; **SPM** – Dryanovo Monastery (410–620 m). Phytophagous, collected from *Parthenocissus* sp. (Popov 1982b).

***Echinothrips americanus* Morgan, 1913**

**Distribution.** Greenhouses in **BS** – Burgas; **PT** – Plovdiv; **PVS** – Sofia. Phytophagous, plant pest of *Chrysanthemum* sp., *Euphorbia* sp., *Hibiscus* sp., *Impatiens* sp., *Syngonium* sp. (Karadjova and Krumov 2003).

***Frankliniella intonsa* (Trybom, 1895)**

**Distribution.** **DM** – Krushovitsa; **PVL** – Lyulin Monastery; **PVP** – Gorni Lozen; **PVS** – Kostinbrod, Svetovrachane; **PVV** – Boyana, Aleko; **ROG** – widespread in Ograzhden Mts.; **RRW** – Trigrad, Smolyan (155–1840 m). Phytophagous, plant pest collected from *Avena sativa*, *Campanula* sp., *Lotus corniculatus*, *Medicago sativa*, *Onobrychis sativa*, *Ranunculus arvensis*, *Trifolium pratense*, *Verbascum* sp., mixed herbaceous vegetation (Genov 1967, Donchev 1968, 1972, Yanev 1968, Yanev 1973, Popov 1982a).

***Frankliniella occidentalis* (Pergande, 1895)**

**Distribution.** Greenhouses in **BS** – Burgas; **PT** – Plovdiv; **PVS** – Sofia; **ROP** – Petrich; **ROT** – Blagoevgrad; **RPM** – Banya. Phytophagous, plant pest of *Alstromeria* sp., *Calla* sp., *Chrysanthemum* sp., *Cucumis sativus*, *Dianthus* sp., *Gerbera jamesonii*, *Gladiolus* sp., *Petunia hybrida*, *Primula* sp., *Rosa* sp., *Saintpaulia ionantha*, *Solanum lycopersicum* (Trenchev 1991, Trenchev and Karadjova 1992).

***Frankliniella pallida* (Uzel, 1895)**

**Distribution.** **DM** – Krushovitsa; **PK** – Breznik valley; **PVV** – Boyana, Dragalevci Simeonovo; ROG – widespread in Ograzhden Mts.; **ROP** – Petrich; **ROT** – Blagoevgrad; **RPR** – Partizanska poliana; **SPM** – Zlatna Panega (155–1500 m). Phytophagous, collected from *Chrysanthemum leucanthemum*, *Coronilla emerus*, *Hypericum perforatum*, *Rumex* sp., *Silene juvenalis*, *Trifolium pratense*, *Xeranthemum* sp., *Viola* sp. mixed herbaceous vegetation (Yanev 1968, Donchev 1976, Popov 1982a).

***Frankliniella tenuicornis* (Uzel, 1895)**

**Distribution.** **DM** – Krushovitsa; **PT** – Pazardzhik; **PVS** – Gorublyane; **PVV** – Dragalevtsi; **RPP** – Delchevo; **ROO** – Kyustendil valley – Bagrentsi (155–1025 m). Phytophagous, collected from *Antirrhinum* sp., *Avena sativa*, *Delphinium* sp., *Hordeum vulgare*, *Medicago sativa*, *Triticum aestivum* (Veselinov 1968, Donchev 1968, 1976).

***Idolimothrips paradoxus* Priesner, 1920**

**Distribution.** **PKG** – Debeli Lag (600 m). Phytophagous, collected from *Bellis perennis* (Krumov 2013).

***Iridothrips mariae* Pelikán, 1961**

**Distribution.** **PVP** – Plana Mts.; **ROG** – valley of river Lebnitsa; **SBW** – Katina (585–1200 m). Phytophagous, collected from *Typha latifolia* (Jenser and Krumov 2009).

***Iridothrips iridis* (Watson, 1924)**

**Distribution.** **DEL** – Kalimok-Brushlen Protected Site (25 m). Hygrophilous and phytophagous, found in the leaf sheaths of *Iris pseudacorus* (Krumov 2013).

***Kakothrips dentatus* Knechtel, 1938**

**Distribution.** **DM** – Krushovitsa; **ROG** – Churicheni, Dolene (155–1000 m). Phytophagous, collected from *Carduus* sp., *Trifolium* sp. mixed herbaceous vegetation (Donchev 1968, Popov 1982a).

***Kakothrips pisivorus* (Westwood, 1880)**

**Distribution.** **DM** – Krushovitsa; **PSL** – Gorni Lozen; **PVV** – Boyana, Cherni vruh, Momina skala, Selimitsa; **ROG** – Divechova polyana (150–2290 m). Phytophagous, collected from *Coronilla varia*, *Lathyrus sativus*, *Lathyrus tuberosus*, *Lepidium draba*, *Lotus corniculatus*, *Medicago sativa*, *Onobrychis sativa*, *Pisum sativum*, *Secale cereale*, *Taraxacum officinale*, *Taraxacum incarnate*, *Trifolium pratense*, *Trifolium repens*, *Vicia faba*, *Vicia sativa* (Genov 1967, Donchev 1968, Yanev1968, Popov 1982a).

***Krokeothrips innocens* (Priesner, 1922)**

**Distribution.** **ROG** – Karnalovo (150–300 m). Phytophagous, collected from mixed grasses (Popov 1982a).

***Limothrips angulicornis* Jablonowski, 1894**

**Distribution.** **ROG** – Churicheni, Karnalovo (150–1000 m). Phytophagous, collected from *Hordeum murinum*, *Hordeum maritimum* (Popov 1982a).

***Limothrips cerealium* Haliday, 1836**

**Distribution.** **PT** – Sadovo; **DEL** – Obrazov Chiflic; **DEP** – Popovo, Tutrakan, Preslav; **DM** – Gorna Oryahovitsa, Veliko Tarnovo); **PVS** – Sofia; **ROG** – Churicheni, Dolene Karnalovo (150–1000 m). Phytophagous, plant pest of *Bromus* sp., *Hordeum* sp., *Hordeum vulgare*, *Pisum sativum*, *Triticum aestivum* (Malkov 1902, Dospevski 1910, Popov 1982a).

***Limothrips consimilis* Priesner, 1926**

**Distribution.** **ROP** – Samuilova krepost (150–300 m). Phytophagous, collected from *Poa* sp. (Popov 1982a).

***Limothrips denticornis* Haliday, 1836**

**Distribution.** **DEP** – Razgrad; **DM** – Chaira, Krushovitsa; **PT** – Sadovo; **PVS** – Sofia,, Kostinbrod, Lokorsko; **PVV** – Aleko, Boyana, Kumata, Malak rezen, Momina skala, Ostrica, Selimitsa, Simeonovo, Trite kladentsi; **ROG** – Karnalovo; RPP – Predela – Gradevo; **RRW** – Smolyan Lakes; **SBM** – Ribaritsa (155–2400 m). Phytophagous, plant pest, collected from *Alopecurus* sp., *Avena sativa*, *Dactylis glomerata*, *Dianthus* sp., *Eriophorum gracile*, *Festuca* sp., *Hordeum vulgare*, *Lotus corniculatus*, *Medicago sativa*, *Pinus montana*, *Poa* sp., *Poa alpina*, *Rubus* sp., *Secale cereale*, *Solanum dulcamara*, *Triticum aestivum*, *Trifolium pratense*, *Vaccinium vitis idea*, mixed herbaceous vegetation (Dospevski 1910, Donchev 1968, 1976, Veselinov 1968, Yanev 1968, 1973, Popov 1982a, 1982b, Krasteva et al. 2013).

***Limothrips schmutzi* Priesner, 1919**

**Distribution.** **PVV** – Boyana, Dragalevtsi, Kaleto, Vladaya, Rudartsi; **RPM** – Banya; **SBW** – Berkovitsa (750–1050 m). Phytophagous, collected from *Alopecurus* sp., *Alopecurus montanum*, *Avena* sp., *Avena sativa*, *Crataegus montania*, *Phleum* sp., *Plantago* sp., *Poa alpina*, *Rosa* sp., *Rubus* sp, *Vaccinium vitis-idaea* (Yanev 1968, Popov 1982b).

***Mycterothrips albidicornis* (Knechtel, 1923)**

**Distribution.** **ROG** – Markovi Kladentsi (1200–1535 m). Phytophagous, collected from leaves of *Fagus sylvatica* (Popov 1982a).

***Mycterothrips consociatus* (Targioni-Tozzetti, 1886)**

**Distribution.** **ROB** – Belasitsa Mts. (600 m). Phytophagous, collected from leaves of *Quercus coccifera* (Popov 1988).

***Mycterothrips latus* (Bagnall, 1912)**

**Distribution.** **ROP** – Struma valley – Kresna; **SPM** – Reselets (165–210 m). Phytophagous, collected from leaves of *Sambucus* sp. (Popov 1982b).

***Mycterothrips salicis* (O. M. Reuter, 1879)**

**Distribution.** **PVS** – Sofia (500–700 m). Phytophagous, collected from leaves of *Tilia* sp. (Popov 1982b).

***Odontothrips confusus* Priesner, 1926**

**Distribution.** **BN** – Obzor; **DEL** – Obrazcov chiflic, Hursovo, Rujitsa; **DM** – Krushovitsa; **PT** – Pazardjik, Plovdiv, Opan; **ROG** – Churicheni; **SPW** – Lilyache (130–300 m). Phytophagous, collected from *Lotus corniculatus*, *Medicago lupulina*, *Medicago sativa*, *Melilotus albus*, *Onobrychis caput-galli*, mixed herbaceous vegetation (Donchev 1968, 1976, Popov 1982a).

***Odontothrips cytisi* Morison, 1928**

**Distribution.** **RPS** – Slavyanka Mts. (720–1170 m). Phytophagous, collected from *Cytisus* sp. (Popov 1988).

***Odontothrips dorycnii* Priesner, 1951**

**Distribution.** **ROP** – Melnik (437 m). Phytophagous, collected from *Dorycnium germanicum* (Jenser and Krumov 2009).

***Odontothrips intermedius* (Uzel, 1895)**

**Distribution.** **PVV** – Momina scala, Planinets (1365–1485 m). Phytophagous, collected from mixed herbaceous vegetation (Yanev 1968).

***Odontothrips loti* (Haliday, 1852)**

**Distribution.** **DM** – Valchitran; **PT** – Stara Zagora; **ROG** – Ograzhden Mts., **RPR** – Borovec, Partizanska poliana; **SBM** – Glozhene; **SPM** – Gorsko Slivovo; **SPW** – Botevgrad (155–1350 m). Phytophagous, collected from *Coronilla emerus*, *Fabaceae* species, *Lathyrus* sp., *Lotus corniculatus*, *Medicago sativa*, mixed herbaceous vegetation (Donchev 1972, 1976, Popov 1982a).

***Odontothrips meliloti* Priesner, 1951**

**Distribution.** **DM** – Krushovitsa; **ROG** – Churicheni, Karnalovo (150–1000 m). Phytophagous, collected from *Melilotus officinalis*, *Melilotus* sp. (Donchev 1968, Popov 1982a).

***Odontothrips meridionalis* Priesner, 1919**

**Distribution.** **ROG** – Churicheni (300–1000 m). Phytophagous, collected from mixed herbaceous vegetation (Popov 1982a).

***Odontothrips phaleratus* (Haliday, 1836)**

**Distribution.** **RPR** – Borovets (1350 m). Phytophagous, collected from *Lathyrus* sp., *Lotus corniculatus*, *Medicago sativa*, *Trifolium* sp. (Donchev 1976).

***Oxythrips ajugae* Uzel, 1895**

**Distribution.** **PVV** – Kumata, Sredec, Zlatnite mostove; **ROG** – Churicheni, Gorski Dom; **RPP** – Dolno Kornichko ezero; **SBW** – Kom peak, Vezhen peak (300–1650 m). Phytophagous, collected from *Campanula alpina*, *Eriophorum gracile*, *Juniperus communis*, *Pinus montana*, *Pinus sylvestris*, *Ranunculus montanum*, *Verbascum pannosum* (Yanev 1968, 1973, Popov 1982a, 1982b).

***Oxythrips bicolor* (O. M. Reuter, 1879)**

**Distribution.** **ROG** – Divechova polyana, Gorski Dom (1000–1250 m). Phytophagous, collected from *Pinus sylvestris* (Popov 1982a).

***Oxythrips ulmifoliorum* (Haliday, 1836)**

**Distribution.** **SBW** – Belidie Han (735 m). Phytophagous, collected from *Syringa vulgaris* (Popov 1982b).

***Prosopothrips vejdovskyi* Uzel, 1895**

**Distribution.** **SBW** – Gintsi (1000 m). Phytophagous, collected from Poaceae (Jenser and Krumov 2009).

***Rhaphidothrips longistylosus* Uzel, 1895**

**Distribution.** **ROG** – Nikudin, Gorski Dom (712–1250 m). Phytophagous, collected from *Bromus mollis* (Popov 1982a).

***Rubiothrips ferrugineus* (Uzel, 1895)**

**Distribution.** **SBM** – Beklemeto (1360 m). Phytophagous, collected from *Galium* sp. (zur Strassen 1996).

***Rubiothrips silvarum* (Priesner, 1920)**

**Distribution.** **ROG** – Churicheni, Divechova polyana (300–1150 m). Phytophagous, collected from mixed vegetation (Popov 1982a).

***Rubiothrips validus* (Karny, 1910)**

**Distribution.** **ROP** – Kresna; **SBW** – Gintsi; (165–1000 m). Phytophagous, collected from Rubiaceae and mixed vegetation (Jenser and Krumov 2009).

***Rubiothrips vitalbae* (Bagnall, 1926)**

**Distribution.** **ROP** – Kresna (165 m). Phytophagous, collected from *Clematis vitalba* (Jenser and Krumov 2009).

***Scolothrips longicornis* Priesner, 1926**

**Distribution.** **RPR** – Yastrebets (2230 m). Predator of mites, collected from leaves of *Genista rumelica* (Donchev 1976).

***Scolothrips uzeli* (Schille, 1910)**

**Distribution.** **ROB** – Belasitsa Mts.; **RPR** – Rila Mts.; (800–1490 m). Predator of mites, collected from *Juniperus communis* (Popov 1988).

***Stenothrips graminum* Uzel, 1895**

**Distribution.** **DM** – Krushovitsa; **PK** – Breznik valley; **PT** – Pazardzhik; **PVS** – Sofia; **ROG** – Ograzhden Mts.; **RPR** – Yastrebets (155–2230 m). Phytophagous, collected from *Avena sativa*, *Medicago sativa*, *Melilotus officinalis*, *Hordeum vulgare*, *Galium* sp., *Onobrychis sativa*, *Phleum pratense*, mixed Poaceae (Donchev 1968, 1976, Popov 1982a).

***Taeniothrips inconsequens* (Uzel, 1895)**

**Distribution.** **PVV** – Aleko, Boerica, Boyana, Konyarnika, Planinets, Selimitsa, Trendafila, Zlatnite mostove; **ROP** – Petrich, Samuilovo; **RPP** – Predela – Gradevo (300–1840 m). Phytophagous, collected from *Ficaria verna*, *Malus sylvestris*, *Mentha* sp., *Pinus montana*, *Pyrus communis*, *Primula* sp., *Prunus dulcis*, *Prunus persica*, *Prunus spinosa*, *Ranunculus aquaticus* (Yanev 1968, Popov 1982, Staneva 1991).

***Taeniothrips picipes* (Zetterstedt, 1828)**

**Distribution.** **DM** – Krushovitsa; **ROG** – Divechova polyana, Nikudin; **ROP** – Samuilova krepost; **RPR** – Partizanska polyana; **SBM** – Beklemeto, Troyan (150–1500 m). Phytophagous, collected from *Coronilla emerus*, *Lotus corniculatus*, *Primula* sp., *Trifolium pratense*, *Verbascum* sp. (Donchev 1972, 1976, Popov 1982a).

***Tamaricothrips tamaricis* (Bagnall, 1926)**

**Distribution.** **ROP** – Kresna Gorge (300–500 m). Phytophagous, collected from *Tamarix* sp. (Popov 1982b).

***Tenothrips croceicollis* (Priesner, 1919)**

**Distribution.** **DM** – Krushovitsa; **RRW** – Studenets (160–1735 m). Phytophagous, collected from *Cichorium intybus*, *Erigeron canadensis*, *Geranium macrorrhizum*, *Hypochaeris radicata*, *Leontodon* sp. *Sonchus arvensis*, *Verbascum* sp. (Donchev 1993).

***Tenothrips discolor* (Karny, 1907)**

**Distribution.** **DM** – Krushovitsa (160 m). Phytophagous, collected from *Lotus corniculatus* (Donchev 1976).

***Tenothrips frici* (Uzel, 1895)**

**Distribution.** **DM** – Krushovitsa; **PSP** – Tazha; **PVS** – Kostinbrod; **RPR** – Musala peak; **SBM** – Teteven; **SBW** – Botevgrad; **SPW** – Lilyache (160–2925 m). Phytophagous, collected from *Carduus* sp., *Dactylis glomerata*, *Lotus corniculatus*, *Medicago sativa*, *Senecio* sp., *Trifolium pratense* (Donchev 1972, 1976).

***Theilopedothrips pilosus* (Uzel, 1895)**

**Distribution.** **ROG** – Dolene, Markovi kladentsi, Divechova polyana (400–1535 m). Phytophagous, collected from mixed herbaceous vegetation (Popov 1982a).

***Thrips albopilosus* Uzel, 1895**

**Distribution.** **RPP** – Yavorov; Predela (1050–1740 m). Phytophagous, collected from *Juniperus communis*, *Juniperus* sp. (Popov 1988).

***Thrips alni* Uzel, 1895**

**Distribution.** **ROP** – Melnik, Kresna Gorge; RPP – Predela – Gradevo (300–500 m). Phytophagous, collected from *Alnus glutinosa*, *Coylus* sp. (Popov 1988).

***Thrips angusticeps* Uzel, 1895**

**Distribution.** **DM** – Krushovitsa, Valchi Tran; **PVV** – Simeonovo; **ROG** – Karnalovo (150–550 m). Phytophagous, collected from *Hordeum vulgare*, *Lotus corniculatus*, *Medicago sativa*, *Onobrychis sativa*, *Sinapis arvensis*, *Triticum aestivum*, mixed herbaceous vegetation (Donchev 1968, 1972, 1976, Veselinov 1968, Popov 1982a).

***Thrips atratus* (Haliday, 1836)**

**Distribution.** **DM** – Krushovitsa; **PBC** – Topolovgrad – Hlyabovo; **ROG** – Ograzhden Mts.; **ROT** – Bobochevo); **SPW** (Botevgrad); **RPR** – (Borovec, Musala peak, Partizanska poliana, Rila); **SPM** – (Zlatna Panega) (155–2925 m). Phytophagous, collected from *Centaurium erythraea*, *Genista tinctoria*, *Haberlea rhodopensis*, *Lotus corniculatus*, *Matricaria chamomilla*, *Medicago sativa*, *Nicotiana tabacum*, *Onobrychis sativa*, *Sorghum halepense*, *Thymus* sp., *Trifolium pratense*, *Trifolium repens*, mixed herbaceous vegetation of *Poaceae*, *Fabaceae* (Chorbadzhiev 1929, Donchev 1976, Popov 1982a).

***Thrips calcaratus* Uzel, 1895**

**Distribution.** **PVS** – Sofia (500 m). Phytophagous, collected from *Tilia* sp. (Popov 1982b).

***Thrips dificilis* Priesner, 1920**

**Distribution.** **PVP** – Kokalyane; **PVS** – Opicvet; **SBM** – Teteven (410–685 m). Phytophagous, collected from *Salix* sp., *Salix babylonica*, *Salix purpurea* (Popov 1982b).

***Thrips dilatatus* Uzel, 1895**

**Distribution.** **ROG** – Divechova polyana, Nikudin (300–1150 m). Phytophagous, collected from mixed herbaceous vegetation (Popov 1982a).

***Thrips discolor* Haliday, 1836**

**Distribution.** **ROG** – widespread in Ograzhden Mts. (100–700 m). Phytophagous, collected from mixed herbaceous vegetation (Popov 1982a).

***Thrips euphorbiae* Knechtel, 1923**

**Distribution.** **ROG** – Karnalovo (150–300 m). Phytophagous, collected from *Euphorbia* sp. (Popov 1982a).

***Thrips fedorovi* (Priesner, 1933)**

**Distribution.** no specific location is mentioned. Phytophagous, collected from *Rosa canina*, *Salvia sclarea* (Schliephake 1983).

***Thrips flavus* Schrank, 1776**

**Distribution.** **PT** – Sadovo; **RPR** – Borovets, Granchar, Partizanska polyana (155–2185 m). Phytophagous, collected from *Lathyrus aureus*, *Hypericum perforatum*, *Verbascum phlomoides* (Manushev 1897, Donchev 1976).

***Thrips fuscipennis* Haliday, 1836**

**Distribution.** ROG – valley of river Lebnitsa (700 m). Phytophagous, collected from *Platanus acerifolia*, *Platanus orientalis* (Jenser and Krumov 2009).

***Thrips italicus* (Bagnall, 1926)**

**Distribution.** **ROG** – Nikudin; **ROP** – Samuilova krepost (150–1000 m). Phytophagous, collected from *Bellis* sp., *Chrysanthemum* sp., *Euphorbia* sp., *Matricaria* sp. (Popov 1982a).

***Thrips juniperinus* Linnaeus, 1758**

**Distribution.** **RPP** – Yavorov, Popina luka; (1250–1740 m). Phytophagous, collected from *Juniperus communis*, *Juniperus* sp. (Popov 1988).

***Thrips linariae* (Priesner, 1928)**

**Distribution.** **RPR** – Partizanska poliana (1500 m). Phytophagous, collected from *Hypericum perforatum*, *Lotus corniculatus*, *Verbascum phlomoides* (Donchev 1976).

***Thrips linarius* Uzel, 1895**

**Distribution.** **DEL** – Dobrudja (230 m). Phytophagous, plant pest, collected from *Agrostemma githago*, *Euphorbia* sp., *Linum usitatissimum*, *Sinapis* sp. (Kirkov 1954).

***Thrips major* Uzel, 1895**

**Distribution.** **ROG** – Churicheni; **ROP** – Samuilova krepost; **RPM** – Banya; **RPR** – Partizanska polyana; **SBW** – Berkovitsa (150–1500 m). Phytophagous, collected from *Alopecurus agrestis*, *Lotus corniculatus*, *Rosa* sp. (Donchev 1976, Popov 1982a, 1982b).

***Thrips mareoticus* (Priesner, 1932)**

**Distribution.** **ROP** – Samuilova krepost (150–300 m). Phytophagous, collected from *Lepidium* sp. (Popov 1982a).

***Thrips meridionalis* (Priesner, 1926)**

**Distribution.** **ROG** – Nikudin; **ROP** – Petrich, Samuilova krepost; **ROT** – Blagoevgrad; **SBM** – Beklemeto, Troyan; **RPR** – Granchar, Smradlivoto ezero, Partizanska polyana (150–2295 m). Phytophagous, plant pest, collected from *Asteraceae*, *Campanula* sp., *Cornus sanguinea*, *Coronilla emerus*, *Euphorbia* sp., *Hieracium* sp., *Lotus corniculatus*, *Genista tinctoria*, *Malus domestica*, *Prunus dulcis*, *Prunus persica*, *Prunus spinosa*, *Ranunculus* sp., *Trifolium repens*, *Verbascum phlomoides* (Donchev 1968, 1976, Popov 1976, 1982a, Staneva 1991).

***Thrips minutissimus* Linnaeus, 1758**

**Distribution.** **ROG** – Markovi kladentsi (1532 m). Phytophagous, collected from mixed herbaceous vegetation (Popov 1985).

***Thrips nigropilosus* Uzel, 1895**

**Distribution.** **PVS** – Gorublyane; **DM** – Komudara (150–550 m) Phytophagous, collected from *Avena sativa*, *Medicago sativa*, *Sorghum halepense* (Veselinov 1968, Donchev 1976).

***Thrips physapus* Linnaeus, 1758**

**Distribution.** **DM** – Krushovitsa; **ROP** – Parvomai; **RPR** – Yastrebetz, Grunchar, Smradlivo ezero, Partizanska poliana; **PSP** – Tazha (150–2295 m). Phytophagous, collected from *Cardus* sp., *Euphorbia* sp., *Genista tinctoria*, *Hypericum perforatum*, *Medicago sativa*, *Senecio* sp., *Solanum tuberosum*, *Viola* sp. (Donchev 1968, 1972, 1976; Popov 1982a).

***Thrips pini* (Uzel, 1895)**

**Distribution.** **ROG** – Churicheni, Divechova polyana; **RPP** – Predela – Gradevo; **SBM** – Vasilyovo (300–1150 m). Phytophagous, collected from *Asteraceae* plants *Pinus* sp., *Pinus sylvestris*, *Picea* sp., *Verbascum* sp. (Popov 1976, 1982a, 1985b).

***Thrips sambuci* Heeger, 1854**

**Distribution.** **ROP** – Kresna Gorge; **SPM** – Reselets (205–500 m). *Picea* sp., *Sambucus* sp. (Popov 1982b).

***Thrips simplex* (Morison, 1930)**

**Distribution.** Greenhoses and open field **PVS** – Negovan (500 m). Phytophagous, collected from Iridaceae (*Gladiolus* sp.) (Donchev 1984).

***Thrips tabaci* Lindeman, 1889**

**Distribution.** widespread in the country, **DEP** – Isperih; **DM** – Chaira, Krushovitsa, Lovech, Pleven; **PVS** – Kostinbrod; **PVV** – Dragalevtsi, Kumata, Malinazha, Selimitsa, Tintyava, Rodina; **ROP** – Melnik, Petrich; ROT – Bobochevo; **RPR** – Rila; **RPM** – Gotce Delchev; **SBM** – Beklemeto, Troyan, Vasilyovo; **PT** – Pazardzhik, Plovdiv, Sadovo, Haskovo (50–2200 m). Phytophagous, plant pest, collected from *Beta vulgaris*, *Dianthus* sp., *Galium* sp., *Hypericum perforatum*, *Ligustrum vulgare*, *Lotus corniculatus*, *Medicago sativa*, *Melilotus officinalis*, *Nicotiana tabacum*, *Onobrychis sativa*, *Poa pratensis*, *Primula elatior*, *Sinapis arvensis*, *Solanum dulcamara*, *Trifolium pratense*, *Vaccinium* sp., *Vaccinium myrtillus*, *Verbascum* sp. (Malkov 1903, Yanev 1968, Donchev 1968, 1972, Popov 1982a, 1982b).

***Thrips trehernei* Priesner, 1927**

**Distribution.** **RPR** – Granchar (2185 m). Phytophagous, collected from *Trifolium repens* (Donchev 1976).

***Thrips urticae* Fabricius, 1781**

**Distribution.** **PT** – Sadovo; **ROP** – Samuilova krepost (150–300 m). Phytophagous, collected from *Nicotiana tabacum*, *Urtica dioica*, *Ranunculus* sp. (Manushev 1897, Popov 1982a).

***Thrips validus* Uzel, 1895**

**Distribution.** **ROG** – widespread in Ograzhden Mts.; **RPR** – Borovets (150–1350 m); Phytophagous, collected from mixed *Asteraceae* plants, *Lathyrus* sp. (Donchev 1976, Popov 1982a).

***Thrips verbasci* (Priesner, 1920)**

**Distribution.** **DM** – Krushovitsa; **PSP** – Tazha; **ROG** – widespread in Ograzhden Mts.; **SBM** – Beklemeto, Troyan; **SBW** – Vezhen (155–1650 m). Phytophagous, collected from *Galium* sp., *Lotus corniculatus*, *Verbascum* sp. (Donchev 1968, 1976, Popov 1982a).

***Thrips viminalis* Uzel, 1895**

**Distribution.** **SPM** – Reselets (210 m). Phytophagous, collected from *Salix* sp. (Popov 1982b).

***Thrips vuiletti* (Bagnall, 1933)**

**Distribution.** **ROG** – Divechova polyana (1000 m). Phytophagous, collected from mixed grasses (Popov 1982a).

***Thrips vulgatissimus* Haliday, 1836**

**Distribution.** **DM** – Komudara; **PVV** – Kikish, Ostrec, Ostritsa, Planinets; **ROG** – widespread in Ograzhden Mts.; **RPR** – Granchar, Musala peak, Partizanska polyana; **RRW** – Chaira, Smolyan; **SBM** – Beklemeto, Troyan (300–2925 m). Phytophagous, collected from *Campanula* sp., *Hypericum perforatum*, *Medicago sativa*, *Sorghum halepense*, *Trifolium repens*, *Verbascum* sp., mixed herbaceous vegetation from Brassicaceae, Rosaceae (Yanev 1968, 1973, Donchev 1968, 1976, Popov 1982a).

## Suborder Tubulifera Haliday

Suborder Tubulifera consists of about 3500 species and 450 genera, placed in the single family Phlaeothripidae and two subfamilies- Idolothripinae and Phlaeothripinae (ThripsWiki 2015). Species in Idolothripinae are considered to feed on fungal spores (Mound and Palmer 1983), while the Phlaeothripidae are considerably diverse with three recognized “lineages”: *Haplothrips*, *Liothrips* and *Phlaeothrips* (Mound and Marullo 1996). The *Haplothrips* lineage is well defined as the tribe Haplothripini (Mound and Minaei 2007). Species in this tribe are often phytophagous but some are predatory on other small arthropods. Although flower-living is relatively unusual among Phlaeothripidae, in the genus *Haplothrips* a large number of species live in the flowers of Asteraceae, Poaceae and Cyperaceae (Mound and Minaei 2007). Members of the *Liothrips* lineage are leaf-feeding, and many of these are associated with the induction of leaf galls. Species in the *Phlaeothrips* lineage are essentially mycophagous, presumably hyphae feeders, and are often associated with dead leaves and branches (ThripsWiki 2015). Some Phlaeothripidae are associated with mosses, and others are predators on mites or on coccids (Mound 2004). Thirty species of 10 genera have been recorded from Bulgaria.

### Family Phlaeothripidae

#### Subfamily Idolothripinae Bagnall

***Bolothrips bicolor* (Heeger, 1852)**

**Distribution.** **ROG** – Gorski Dom (1250 m). Mycophagous-spore feeder, collected from fallen leaves (Popov 1982a).

***Bolothrips dentipes* (O. M. Reuter, 1880)**

**Distribution.** **ROG** – Churicheni (300–1000 m). Mycophagous-spore feeder, found in soil from a field with *Hordeum vulgare* (Popov 1982a).

***Compsothrips albosignatus* (Reuter, 1884)**

**Distribution.** **ROG** – Markovi kladentsi (1520 m). Mycophagous-spore feeder, collected from *Fagus* sp. (Popov 1982a).

***Cryptothrips nigripes* (Reuter, 1880)**

**Distribution.** **PVL** – Lyulin Monastery; **RRW** – Trigrad; **PVV** – Selimitsa (1000–1300 m). Mycophagous-spore feeder, collected from *Corylus avellana* leaves, and on mixed herbaceous vegetation in oak forests (Yanev 1973).

#### Subfamily Phlaeothripinae Uzel

***Amphibolothrips knechteli* (Priesner, 1936)**

**Distribution.** **BN** – Cape Kaliakra (70 m). Mycophagous -hyphae feeder, found in leaf litter (Vasiliu-Oromulu 1981).

***Cephalothrips monilicornis* (O. M. Reuter, 1880)**

**Distribution.** **BS** – Dyuni (50 m). Unknown feeding preferences, collected from mixed herbaceous vegetation (Jenser and Krumov 2009).

***Haplothrips acanthoscelis* (Karny, 1910)**

**Distribution.** **DM** – Krushovitsa; **PVS** – Kostinbrod; **SPW** – Botevgrad (155–400 m). Phytophagous, collected from *Lotus corniculatus*, *Onobrychis sativa*, *Zea mays* (Popov 1973, Donchev 1976).

***Haplothrips aculeatus* (Fabricius, 1803)**

**Distribution.** **DM** – Krushovitsa; **PBC** – Topolovgrad; **PBB** – Yambol; **PT** – Pazardzhik; **PSL** – Gorni Lozen; **PVS** – Negovan; **PVV** – Simeonovo; **SBM** – Beklemeto, Troyan (155–1360 m). Phytophagous, collected from *Avena sativa*, *Dactylis glomerata*, *Lotus corniculatus*, *Medicago sativa*, *Onobrychis sativa*, *Secale cereale*, *Trifolium repens*, *Trifolium pratense*, *Triticum aestivum* (Genov 1967, Veselinov 1968, Donchev 1976).

***Haplothrips angusticornis* Priesner, 1921**

**Distribution.** **DM** – Krushovitsa; **PBC** – Topolovgrad – Hlyabovo; **PVS** – Negovan; **ROG** – Ograzhden Mts.; **SPM** – Draganovo; **SPW** – Botevgrad (150–700 m). Phytophagous, collected from *Berberis vulgaris*, *Dactylis glomerata*, *Lotus corniculatus*, *Matricaria chamomilla*, *Medicago sativa*, *Onobrychis sativa*, *Secale cereale*, *Trifolium pratense* mixed grasses (Genov 1967; Veselinov 1968; Donchev 1976; Popov 1982a).

***Haplothrips biroi* (Priesner, 1928)**

**Distribution.** **DM** – Krushovitsa (160 m). Phytophagous, collected from *Lamium purpureum* (Donchev 1993).

***Haplothrips dianthinus* Priesner, 1924**

**Distribution.** **RRW** – Smolyan Lakes (1525 m). Phytophagous, collected from *Dianthus* sp. (Donchev 1993).

***Haplothrips distinguendus* (Uzel, 1895)**

**Distribution.** **ROG** – Nikudin; **ROP** – Samuilova krepost (150–1000 m). Phytophagous, collected from mixed herbaceous vegetation of Asteraceae (Popov 1982a).

***Haplothrips flavicinctus* (Karny, 1910)**

**Distribution.** **DEP** – Makariopolsko; **DM** – Krushovitsa (160–250 m). Phytophagous, collected from *Beta vulgaris*, *Medicago sativa*, *Onobrychis sativa* (Donchev 1968, 1976).

***Haplothrips hispanicus* Priesner, 1924**

**Distribution.** **PBC** – Topolovgrad – Hlyabovo (400 m). Phytophagous, collected from *Haberlea rhodopensis* (Donchev 1976).

***Haplothrips leucanthemi* (Schrank, 1781)**

**Distribution.** **DM** – Krushovitsa; **PBS** – Kosti; **PVS** – Chepintsi, Filipovtsi, Trebich; **ROG** – widespread in Ograzhden Mts.; **RPP** – Dolnoto breznichko ezero **SBM** – Beklemeto, Teteven, Troyan (50–1965 m). Phytophagous, collected from mixed herbaceous of *Asteraceae*, *Medicago sativa*, *Trifolium pratense*, *Trifolium repens* (Donchev 1968, 1976, Yanev 1973, Popov 1982a).

***Haplothrips marrubiicola* Bagnall, 1932**

**Distribution.** **DM** – Krushovitsa (160 m). Phytophagous, collected from *Onobrychis sativa* (Donchev 1976).

***Haplothrips minutus* (Uzel, 1895)**

**Distribution.** **RPR** – Vada hut (1410 m). Phytophagous, collected from shrubby vegetation (Yanev 1973).

***Haplothrips phyllophilus* Priesner, 1914**

**Distribution.** **SBM** – Ribarica (600 m). Phytophagous, collected from *Fagus sylvatica* (Popov 1982b)

***Haplothrips propinquus* Bagnall, 1933**

**Distribution.** **RPR** – Partizanska polyana (1500 m). Phytophagous, collected from *Achilea millefolium*, *Onobrychis sativa* (Donchev 1976).

***Haplothrips reuteri* (Karny, 1907)**

**Distribution.** **DEL** – Obrazov chiflic; **DEP** – Razgrad; **DM** – Krushovitsa; **DW** – Boychinovci; **PBC** – Topolovgrad – Hlyabovo; **PSL** – Gorni Lozen; **PSP** – Sliven; **SPM** – Pravec, Zlatna Panega; **PVS** – Suchodol, Kazichene, Gorna Banya; **RPR** – Granchar (155–2200 m). Phytophagous, collected from *Centaurea cyanus*, *Dactylis glomerata*, *Haberlea rhodopensis*, *Helianthus annuus*, *Medicago sativa*, *Onobrychis sativa*, *Secale cereale*, *Senecio* sp., *Sorghum* sp., *Trifolium repens*, *Triticum aestivum*, *Zea mays* (Chorbadzhiev 1929, Donchev 1968, 1976, Yanev 1973).

***Haplothrips scythicus* Knechtel, 1961**

**Distribution.** **DM** – Krushovitsa (160 m). Phytophagous, collected from *Medicago sativa* (Donchev 1976).

***Haplothrips setiger* Priesner, 1921**

**Distribution.** **DM** – Krushovitsa; **SPW** – Botevgrad; **PBC** – Topolovgrad – Hlyabovo; **ROG** – Nikudin; **ROP** – Parvomai; **RPR** – Borovets, Granchar, Musala peak, Yastrebetz (150–2925 m). Phytophagous, collected from *Aster junceus*, *Chirothrips cinerariifolium*, *Euphorbia* sp., *Genista tinctoria*, *Haberlea rhodopensis*, *Inula helenium*, *Lathyrus* sp., *Lotus corniculatus*, *Melilotus albus*, *Trifolium pratense*, *Trifolium repens*, *Thymus* sp., *Viola* sp., mixed herbaceous vegetation (Donchev 1976, Popov 1973, 1982a).

***Haplothrips subtilissimus* (Haliday, 1852)**

**Distribution.** **SPM** – Pravec (405 m). Phytophagous and facultative predator, collected from *Haberlea rhodopensis* (Donchev 1976).

***Haplothrips tritici* (Kurdjumov, 1912)**

**Distribution.** **DM** – Pavlikeni, Gorna Oryahovitsa; **DW** – Boychinovci; **PSP** – Sliven; **PVS** – Kazichene, Kostinbrod, Prolesha, Svetovrachane; **ROG** – widespread in Ograzhden Mts. (300–1000 m). Phytophagous, pest of cereals, collected from *Hordeum vulgare*, *Secale cereale*, *Triticum aestivum*, mixed grasses (Chorbadzhiev 1929, Yanev 1973, Popov 1982a, Krasteva et al. 2013).

***Haplothrips verbasci* (Osborn, 1896)**

**Distribution.** **PVS** – Vrana; **ROG** – widespread in Ograzhden Mts.; (200–1000 m). Phytophagous, collected from *Verbascum* sp., *Verbascum thapsus* (Popov 1973, 1982a).

***Hoplothrips semicaecus* (Uzel, 1895)**

**Distribution.** **PKQ** – Blateshnitsa (800 m). Mycophagous- hyphae feeder on dead tree branches, collected from the leaves of *Clematis vitalba* (Yanev 1973).

***Hoplothrips ulmi* (Fabricius, 1781)**

**Distribution.** **PVS** – Bankia, Suhodol, Lokorsko (585–695 m). Mycophagous -hyphae feeder on dead parts, large branches, found in the field with *Avena sativa*, mixed herbaceous vegetation in pine forests (Yanev 1973).

***Liothrips pragensis* Uzel, 1895**

**Distribution.** **RPP** – Predela – Gradevo (500 m). Phytophagous, collected from *Quercus sessile* leaves (Popov 1976).

***Phlaeothrips coriaceus* Haliday, 1836**

**Distribution.** **RPR** – Vada hut; **RRW** – Chaira dam (1300–1450 m). Mycophagous- hyphae feeder on dead branches, collected from shrubs in beech forests and meadow vegetation in pine forests (Yanev 1973).

***Xylaplothrips fuliginosus* (Schille, 1911)**

**Distribution.** **RRW** – Smolyan Lakes, Golyam Beglik reservoar; **SBM** – Ribarica; **SPM** – Reselets (210–1600 m). Predator of mites and hyphae feeder, collected from *Populus* sp., shrubs and herbaceous vegetation (Yanev 1973, Popov 1982b).

## Discussion

In total, 155 species of thrips have been recorded in Bulgaria, in the altitudinal range from 0 to 2925 m a.s.l. Considering the assumption of Hubenov (1996) that there should be about 250 species in the country, order Thysanoptera has been insufficiently studied and research has uncovered merely 60% of its diversity. Currently thrips account for 0.53% of the total number of hexapods reported for Bulgaria.

Two species, *Rubiothrips vitis* and *Hoplothrips pallicornis*, have been reported for Bulgaria in Fauna Europea but there is no actual evidence of their presence in the country and they have not been included in the list. The inconsistency of the information on *Rubiothrips vitis* probably stems from the fact that Bournier (1976) lists *Rubiothrips vitis* as a pest of vines in Bulgaria, quoting Zinca (1964). However, the paper of Zinca does not give any information on the presence of this species in the country. No information on the presence of *Hoplothrips pallicornis* in Bulgaria was found in the literature. The only reference for this species from Europe is the redescription of Priesner (1964) resulting from its interception by New York harbour quarantine. The author explains that *Hoplothrips pallicornis* is found in New York under bark of *Juglans regia* but originally comes from former Yugoslavia, suggesting that it may have a wider distribution at its origin. The authors of the present paper sent an informal request to Fauna Europea to ask for the source of the information leading to the inclusion of *Hoplothrips pallicornis* in the list. The reply was that the only written reference of the species’ presence in Europe is Preisner (1964) but it may be in the extensive collection of Pelikan (pers. comm., Bert Vierbergen, Andrea Hastenpflug-Vesmanis, 4 March, 2015) without ever having been published.

As regards the feeding preferences, 136 (87.7%) of the thrips species present in Bulgaria are phytophagous. The majority of them belong to the largest thysanopteran family, Thripidae (101). All seven reported species from family Melanthripidae are plant feeders. In family Aeolothripidae, there are eight phytophagous species which are also facultative predators. Family Phlaeothripidae includes 21 phytophagous species: 1 from genus *Liothrips* and 20 from genus *Haplothrips*. *Hoplothrips subtilissimus* is also a facultative predator. Seven obligate predators from two families, Aeolothripidae (5) and Thripidae (2), have been reported. All 9 mycophagous thrips species present in Bulgaria belong to the Phlaeothripidae. Four of them are spore feeders (Idolothripinae) and 5 are hyphae feeders, of which *Xylaplothrips fuliginosus* is also a predator on mites. Three thrips species are with unknown feeding preferences.

Fourteen members of the phytophagous group are considered pests on agricultural crops. Among them, *Frankliniella occidentalis* and *Thrips tabaci* have economic importance as pests and vectors of Tomato spotted wilt virus (TSWV) (Karadjova and Krumov 2008), while *Haplothrips tritici* can cause significant damage to cereal crops (Krasteva et al. 2013).

**Figure 1.**
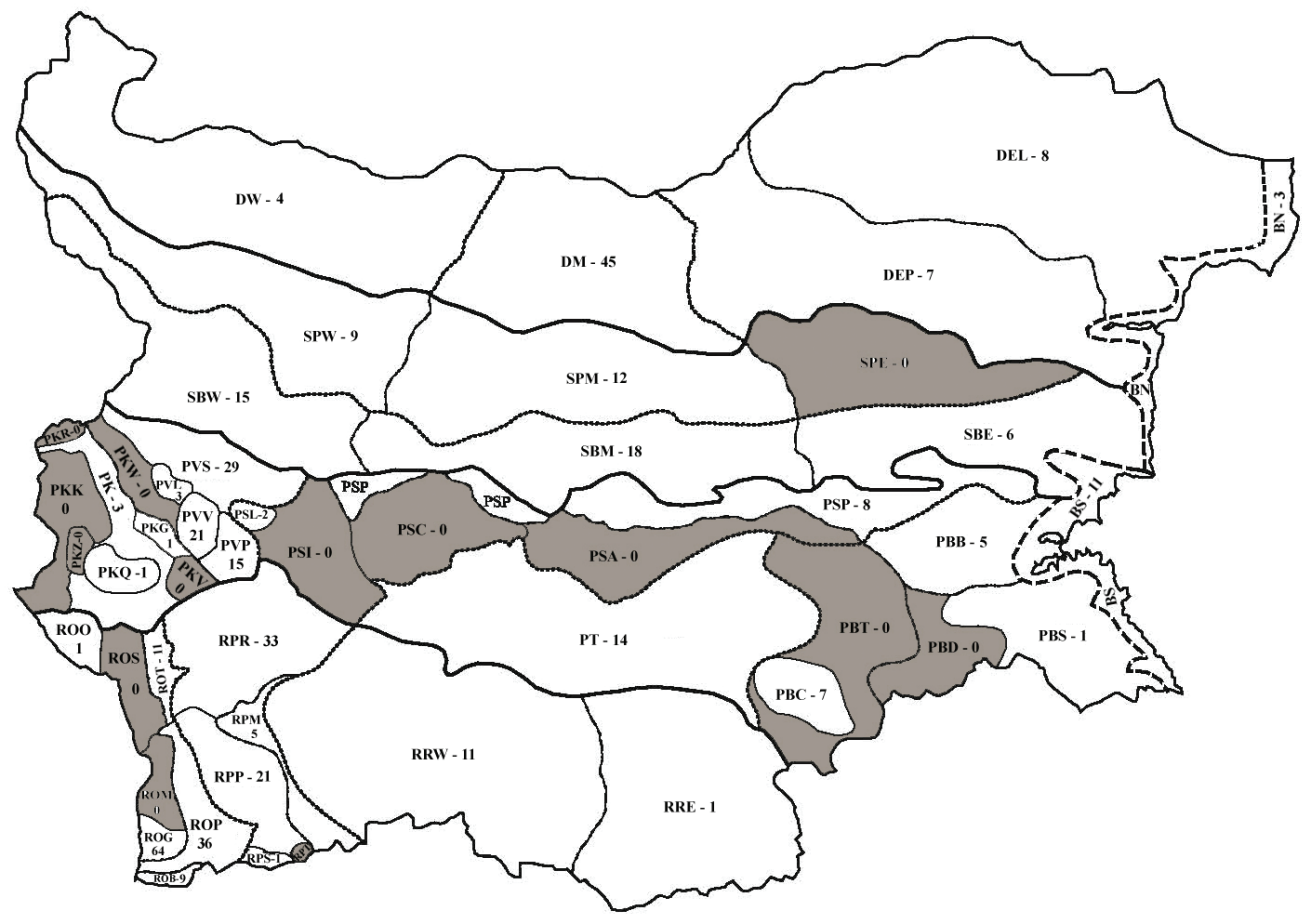
Number of Thysanoptera species found in zoogeographical regions and subregions of Bulgaria.

On Figure 1 the geographical regions and subregions of Bulgaria are presented following the division of Hubenov (1997) and the numbers of thrips species found in each subregion.

Thirteen thrips species have been reported for the region of the Black Sea Coast (B). On the territory of Bulgaria, *Amphibolothrips knechteli* has been found only in the Northern Black Sea Coast subregion (BN), while *Cephalothrips monilicornis*, *Melanthrips knechteli* and *Ropotamothrips buresi* have been recorded only in the Southern subregion (BS) at altitudes close to 0 m a.s.l.

Forty nine species have been reported from the region of the Danubian Plain (D). A single species, *Pseudodendrothrips mori*, has been reported in Bulgaria only from its Western subregion (DW). The Middle subregion (DM) is well studied compared to the rest of the Danubian Plain due to the extensive research of the Bulgarian thysanopterologist Donchev during the period 1968–1996. Four species, *Aeolothrips albicinctus*, *Haplothrips biroi*, *Hoplothrips marrubiicola* and *Tenothrips discolor*, have only been reported from there, at an altitude of 155 m a.s.l. *Iridothrips iridis* and *Thrips linarius* have been reported in Bulgaria only from the Eastern subregion (DE).

The large subregion of the Predbalkan (SP) is scarcely investigated with a total of 18 reported species. In Bulgaria, *Haplothrips subtilissimus* and *Thrips viminalis* are only found in the middle part of the Predbalkan (SPM).

Thirty nine species are found in the region of Stara Planina (Balkan) Mts (SB). *Oxythrips ulmifoliorum* and *Prosopothrips vejdovskyi* have been reported in Bulgaria only for its Western subregion (SBW), while *Haplothrips phyllophilus* and *Rubiothrips ferrugineus* have only been reported from its Middle subregion (SBM).

The parts of the Transitional Region (P) have been investigated to different degrees and are considered separately.

Twelve species have been reported from the Tundja–Strandja Subregion (PB). Currently, only *Haplothrips hispanicus*, found in the Sakar Mts (PBC), is reported in Bulgaria only from this subregion.

The diversity of thrips in the Thracian Lowland (PT) includes 14 species, none of which are found only in this subregion.

A total of nine species have been established in the vast Srednogorie–Podbalkan Subregion (PS) with no records which are unique for the country. Therefore its thysanopteran fauna is almost unknown.

The investigations in Kraishte–Konyavo District (PK) have led to the report of 5 thrips species. *Hoplothrips semicaecus* and *Idolimothrips paradoxus* are only found in Konyavska Planina Mts. (PKQ) and Golo Bardo (PKG), respectively.

The thrips of Vitosha District (PV) are better studied with 52 established species. The following have not been found elsewhere on the territory of Bulgaria: *Aeolothrips priesneri* in the Plana Mts. (PVP); *Aptinothrips elegans*, *Chirothrips aculeatus*, *Hoplothrips ulmi*, *Mycterothrips salicis*, *Thrips calcaratus* and *Thrips simplex* in the Sofia Basin (PVS); *Belothrips morio* and *Odontothrips intermedius* in the Vitosha Mts (PVV).

The Osogovo–Belasitsa region (RO) is the best studied area of Bulgaria, mainly due to the extensive research of T. Popov in Ograzhden during the period 1982–1988. A total of 89 thrips species have been reported, 27 of which have been recorded in Bulgaria only from this region: *Mycterothrips consociatus* in Belasitsa Mts (ROB); *Aeolothrips vittatus*, *Asphodelothrips croceicollis*, *Bolothrips bicolor*, *Bolothrips dentipes*, *Bregmatothrips dimorphus*, *Compsothrips albosignatus*, *Krokeothrips innocens*, *Limothrips angulicornis*, *Mycterothrips albidicornis*, *Odontothrips meridionalis*, *Rubiothrips silvarum*, *Sericothrips bicornis*, *Theilopedothrips pilosus*, *Thrips dilatatus*, *Thrips discolor*, *Thrips euphorbiae*, *Thrips fuscipennis* and *Thrips vuiletti* in Ograzhden Mts (ROG); *Aeolothrips gloriosus*, *Dendrothrips phillireae*, *Odontothrips dorycnii*, *Oxythrips bicolor*, *Rubiothrips validus*, *Rubiothrips vitalbae*, *Tamaricothrips tamaricis*, *Thrips minutissimus* in Krupnik–Sandanski–Petrich Valley (ROP).

Fifty-four species are known from the mountanous Rila–Pirin region (RP). The following are specific only for this region: *Aeolothrips balati*, *Thrips juniperinus*, *Liothrips pragensis* in Pirin Mts. (RPP) with altitudinal range from 400 to 1700 m a.s.l.; *Haplothrips minutus*, *Haplothrips propinquus*, *Odontothrips phaleratus*, *Scolothrips longicornis*, *Thrips linariae*, *Thrips trehernei* in Rila Mts. (RPR) at altitudes ranging from 1350–2230 m a.s.l.; *Odontothrips cytisi* is in Slavyanka Mts. (RPS).

The other large southern mountaineous area of the Rhodope Mts. (RR) is poorly studied with 12 recorded thrips species. *Aptinothrips stylifer*, *Chirothrips pallidicornis* and *Haplothrips dianthinus* are currently known only from the Western Rhodope Mts (RRW).

Three of the species in the list, *Frankliniella occidentalis*, *Echinothrips americanus* and *Heliothrips haemorrhoidalis*, have been reported only from greenhouses.

The number of Thysanoptera species recognized from Bulgaria demonstrates that they constitute one of the not very well studied orders of insects. The Bulgarian Thysanoptera represents less than 1% (0.53%) of the Hexapoda living in Bulgaria.

The reported thysanopteran species from Bulgaria are distributed in the altitudinal range from 0 to 2925 m a s l.
